# Bedürfnisse und Belastungen von palliativmedizinisch mitbetreuten Patienten mit fortgeschrittenen und/oder metastasierten Kopf-Hals-Tumoren

**DOI:** 10.1007/s00106-020-00888-z

**Published:** 2020-05-19

**Authors:** C. Roch, P. Schendzielorz, A. Scherzad, B. van Oorschot, M. Scheich

**Affiliations:** 1 Interdisziplinäres Zentrum Palliativmedizin, Universitätsklinikum Würzburg, Josef-Schneider-Str. 11, 97080 Würzburg, Deutschland; 2 Klinik und Poliklinik für Hals‑, Nasen- und Ohrenkrankheiten, plastische und ästhetische Operationen, Universitätsklinikum Würzburg, Würzburg, Deutschland

**Keywords:** Spezialisierte Palliativversorgung, Screening, Vorsorgedokumente, Qualitätsindikator, stationäre Patienten, Specialised palliative care, Screening, Advance directives, Quality Indicator, Hospitalised patients

## Abstract

**Hintergrund:**

Neue Therapieformen ermöglichen auch bei Patienten mit fortgeschrittenen und metastasierten Kopf-Hals-Tumoren gute Behandlungsansätze. Gespräche über das Lebensende, vorausschauende Versorgungsplanung und auch palliativmedizinische Konzepte geraten dadurch oft in den Hintergrund.

**Ziel der Arbeit:**

Analyse von Symptomen, Belastungen sowie dem Vorliegen von Vorsorgedokumenten, von stationär palliativmedizinisch mitbetreuten Patienten durch Selbsteinschätzung. Erhebung der Integration der spezialisierten Palliativversorgung in die Versorgung stationär verstorbener Patienten anhand des Qualitätsindikators nach Earle.

**Material und Methoden:**

Retrospektive Analyse der vom Palliativdienst mitbetreuten Patienten mit Kopf-Hals-Tumoren durch Nutzung des standardisierten IPOS Fragebogens.

**Ergebnisse:**

Die häufigsten physischen Symptome waren „Schwäche“ (77 %), „Appetitlosigkeit“ (65 %) und „eingeschränkte Mobilität“ (65 %). „Schmerzen“ wurden von 42 % der Patienten angegeben. Die häufigsten emotionalen und psychosozialen Belastungen waren „Traurigkeit“ (97 %) und „Sorgen des Patienten“ (94 %), die „fehlende Möglichkeit Gefühle“ zu teilen (77 %), „unzureichende Informiertheit“ (85 %) und „organisatorische Probleme“ (77 %). Bei 23 % der Patienten lagen Vorsorgedokumente vor. Das Qualitätsziel nach Earle wurde nicht erreicht.

**Diskussion:**

Die Patienten berichten in der Auswertung in hohem Maß Symptome und Belastungen, am häufigsten im emotionalen Bereich und bezüglich kommunikativer und praktischer Bedürfnisse. Hier zeigt sich möglicherweise auch ein Gesprächsbedarf über Wünsche und Vorstellungen im Hinblick auf das Lebensende. Palliativdienste sollten deshalb besonders auf die psychosozialen Bedürfnisse einzugehen.

## Abstract (en)

**Background:**

New therapeutic modalities enable good treatment options for patients with advanced and metastatic head and neck cancer. In this context, discussions about the end of life, advance care planning, and palliative concepts are often eclipsed.

**Objective:**

Analysis of symptom burden and advance directives of palliatively comanaged inpatients using a palliative care self-assessment questionnaire. Based on the Earle quality indicator (QI), the integration of specialized palliative care in deceased inpatients was examined.

**Patients and methods:**

Retrospective analysis of head and neck cancer patients cotreated by the palliative care service. Patients were surveyed using the Integrated Palliative Care Outcome Scale (IPOS) questionnaire.

**Results:**

The most frequent physical symptoms were weakness (77%), loss of appetite (65%), and poor mobility (65%). Pain was reported by 41% of patients. The most frequent emotional and psychosocial burdens were depression (97%) and worries about illness or treatment (94%), lack of ability to share feelings (77%), lack of information (85%), and organizational problems (77%). In 23% of patients, advanced directives were available. The Earle QI was not achieved.

**Discussion:**

Patients report a high degree of symptoms and stress, most often in emotional areas and in terms of communicative and practical needs. This may reflect a need to talk about wishes and expectations concerning the end of life. Palliative care services should aim to address psychosocial needs in particular.

## Hintergrund

Kopf-Hals-Tumoren sind mit 6 % aller Tumoren die sechsthäufigste Tumorentität [1]. Diese Tumoren sind oft bei Diagnosestellung bereits fortgeschritten und nach dem Auftreten von Rezidiven meist nicht mehr kurativ zu behandeln [2]. Aufgrund der langwierigen Therapie und des Tumorprogresses leiden diese Patienten nahe dem Lebensende nicht selten an den sichtbaren Folgen der Tumorerkrankung wie Tracheostomie, perkutane endoskopische Gastrostomie (PEG), Tumorkachexie oder auch Geruchsbelästigung durch Tumorzerfall [2, 3]. Durch Neuzulassungen, Weiterentwicklungen und die verbesserte Verfügbarkeit zielgerichteter medikamentöser Therapien ist es vermehrt möglich, auch Patienten mit schlechtem Allgemeinzustand und fortgeschrittenen Tumoren eine palliative Therapie anzubieten [4]. Der Fokus dieser zielgerichteten Therapien liegt insbesondere in der Hoffnung, Lebenszeit zu gewinnen und den Tumorprogress aufzuhalten [3]. Gespräche über das Lebensende und damit verbundene Wünsche und Notwendigkeiten finden daher häufig erst spät oder gar nicht statt [3].

Um bei Patienten die persönlichen Bedürfnisse besser zu erkennen, wird die Erhebung von „patient-reported outcome measurements“ (PROM) empfohlen [5]. Die S3-Leitlinie Palliativmedizin für nicht heilbare Krebspatienten empfiehlt hierzu unter anderem die Verwendung der multidimensionalen Integrated Palliative Care Outcome Scale (IPOS) [6]. Diese ist eine verbesserte Version der Palliative Care Outcome Scale [7] und umfasst 17 Fragen aus den Bereichen physischer und emotionaler Symptome ebenso wie kommunikativer, spiritueller und praktischer Probleme [8]. Sorgen und Bedürfnisse des Patienten lassen sich hiermit bereits niederschwellig detektieren und können somit direkt angesprochen werden.

Auch Gespräche über die Versorgungsmöglichkeiten am Lebensende sollten frühzeitig offen diskutiert werden [9–11]. Von besonderer Bedeutung sind in diesem Zusammenhang jede Form von Vorsorgedokumenten wie eine Vorsorgevollmacht (VV), eine Betreuungsverfügung (BV) und eine Patientenverfügung (PV), eventuell ergänzt um einen Notfallplan für Krisensituationen. In der VV wird geregelt, wer z. B. den Patientenwillen umsetzen darf, wenn der Patient dazu selbst nicht mehr in der Lage ist, oder wem gegenüber der behandelnde Arzt von seiner Schweigepflicht entbunden ist. In der BV regelt der Patient, wer ggf. als Betreuer gerichtlich bestellt werden soll. Eine sinnvolle Ergänzung zur VV ist die PV. Hier kann der Patient Wünsche für unterschiedliche Lebenssituationen äußern. Beim einwilligungs- oder äußerungsunfähigen Patienten ist die Patientenverfügung ein wertvolles Dokument für den Vorsorgebevollmächtigten und den Arzt im Hinblick auf medizinische Entscheidungen am Lebensende. Die Vorsorgedokumente müssen nicht notariell beurkundet werden. Die PV setzt die Einwilligungsfähigkeit voraus, für eine BV und eine über die gesundheitlichen Angelegenheiten hinausgehende VV ist die Geschäftsfähigkeit erforderlich [12].

Bei Patienten, bei denen eine mögliche tumorbedingte Notfallsituation wie Blutung oder Ersticken vom Primärbehandler so eingeschätzt wird, dass auch eine Maximaltherapie das Versterben nicht verhindern kann, wird eine krankheitsspezifische Erörterung der Behandlungswünsche am Lebensende durchgeführt. Hierzu geht die Klärung realistischer Therapieziele durch den Arzt voraus. Diese stellen eine wesentliche Grundlage der Arzt-Patienten-Beziehung dar und werden in einem Notfallplan festgehalten [13]. Dieser enthält palliative ärztliche Anweisungen zur Symptomlinderung für den vor Ort behandelnden Arzt, der den Patienten möglicherweise nicht kennt. Ebenso kann der Patient fixieren, ob er ausschließlich eine Symptomlinderung vor Ort/zu Hause wünscht oder ob er zur Symptomlinderung in ein Krankenhaus gebracht werden möchte. Weiterhin kann der Patient festlegen, ob er eine intensivmedizinische Behandlung wünscht, falls diese indiziert ist.

Zur Evaluation der Integration der spezialisierten Palliativversorgung (SPV) in die Patientenversorgung am Lebensende definierte Earle 2005 Qualitätsindikatoren [14], die aus Versorgungsdaten generierbar sind. In der Annahme, dass ein sehr später Einbezug der SPV die individuelle Sterbebegleitung erschwert, wurden Qualitätsziele definiert. Diese besagen unter anderem, dass mindestens 55 % der an Krebs Verstorbenen Kontakt zur Palliativmedizin haben sollten und weniger als 8 % dieser Patienten den Kontakt mit der SPV erst innerhalb der letzten 3 Tage vor ihrem Tod haben sollten (Earle-QI).

Am Universitätsklinikum Würzburg gibt es neben einer 10-Betten-Palliativstation seit 2012 einen Palliativdienst (PD), der für die Mitbetreuung der Patienten in den anderen Kliniken zur Verfügung steht. Seit 2018 wird der IPOS routinemäßig in allen Fachdisziplinen beim Erstkontakt mit dem PD zur Patientenselbsteinschätzung genutzt.

In einer retrospektiven Analyse wurden folgende Fragestellungen bearbeitet:

- Über welche Symptome und Belastungen berichten Patienten mit Kopf-Hals-Tumoren beim Erstkontakt mit dem Palliativdienst?
- Wie viele Patienten hatten beim Erstkontakt mit dem PD bereits Vorsorgedokumente?
- In welchem Umfang lagen diese bei Abschluss der Mitbetreuung vor?
- Wird der Earle-Qualitätsindikator erreicht?

## Methoden

### Teilnehmer und Datenerhebung

Aus der elektronischen Patientenakte wurden retrospektiv alle volljährigen Patienten mit Kopf-Hals-Tumoren identifiziert, die zwischen 01.01.2018 und 31.10.2019 vom klinikinternen Palliativdienst (PD) mitbetreut wurden. Hierzu wurden die ICD(Internationale Klassifikation der Krankheiten)-Nummern C00–C14 (bösartige Neubildungen der Lippe, des Zungengrunds, der Zunge, des Zahnfleisches, des Mundbodens, des Gaumens, des Mundes, der Parotis, der sonstigen großen Speicheldrüsen, der Tonsille, des Oropharynx, des Nasopharynx, des Recessus piriformis, des Hypopharynx und nicht näher bezeichneter Lokalisation der Lippe, der Mundhöhle und des Pharynx) sowie C30–C32 (bösartige Neubildungen der Nasenhöhle und des Mittelohrs, der Nasennebenhöhlen und des Larynx) analysiert.

Von diesen Patienten wurde die Diagnose, die Dauer der Erkrankung, das Alter, das Geschlecht, der Allgemeinzustand (Eastern Cooperative Oncology Group, ECOG) und das Tumorstadium bei Zuweisung (Union for International Cancer Control, UICC) an den PD erfasst. Des Weiteren wurde dokumentiert, ob ein Tracheostoma vorhanden war und ob es Vorsorgedokumente in den Unterlagen gab.

### IPOS (Integrated Palliative Care Outcome Scale)

Bei allen Patienten wurde im Rahmen der palliativmedizinischen Anamnese routinemäßig der IPOS erfasst. In die Auswertung der patientengeäußerten Bedürfnisse wurden nur Patienten mit vollständig ausgefülltem IPOS einbezogen. Die Belastung durch die Symptome wird im IPOS auf einer Likert-Skala von 0–4 (0 = keine Probleme, 1 = Symptom mit geringer Ausprägung, 2 = Symptom mit mäßiger Ausprägung, 3 = Symptom mit starker Ausprägung und 4 = Symptom mit sehr starker Ausprägung) angegeben. Es werden Symptome aus 3 Teilbereichen erhoben, hierzu gehören physische Symptome sowie psychische und organisatorisch/kommunikative Belange. Die Messung der physischen Symptome erfolgt als Intensität (0 = gar nicht, 1 = ein wenig, 2 = mäßig, 3 = stark, 4 = extrem stark), alle anderen Symptome werden als Häufigkeiten erfasst (0 = gar nicht, 1 = selten, 2 = manchmal, 3 = meistens, 4 = immer) mit einer Umkehr der Häufigkeiten (0 = immer, 1 = meistens, 2 = manchmal, 3 = selten, 4 = gar nicht) bei den Fragen nach dem „inneren Frieden“, dem „Teilen von Gefühlen“ und der „Informiertheit“. Hohe Punktwerte gehen somit immer mit einem hohen Maß an Belastung einher. In der hier verwendeten Patientenversion werden die Symptome der letzten 3 Tage erfragt.

Nach Murtagh et al. [6] wurde ein Bedürfnis dann als klinisch relevant definiert, wenn es in einer Ausprägung ≥2 (mäßig, schwer, sehr schwer) vorlag.

### Earle-Qualitätsindikator

Die Erhebung des Earle-QI erfolgte anhand der im klinischen Dokumentationssystem erfassten Sterbedaten für die palliativmedizinisch mitbetreuten Patienten. Diese wurden mit dem Datum des Erstkontakts zum PD verglichen. Der Earle-QI wird erreicht, wenn der Anteil an Patienten, die den ersten Kontakt zum PD erst innerhalb der letzten 3 Tage vor dem Tod hatten, unter 8 % liegt und wenn 55 % der Verstorbenen überhaupt Kontakt zur Palliativmedizin hatten.

### Statistik

Die Ergebnisdarstellung erfolgt ausschließlich deskriptiv.

### Datenanalyse

Die statistischen Analysen wurden mit IBM SPSS Statistics 25 (Fa. SPSS Inc., Chicago/IL, USA) durchgeführt.

### Ethik und Datenschutz

Unter der Nummer 20190522 01 wurde eine Bestätigung der Ethikkommission des Universitätsklinikums Würzburg (UKW) für die anonymisierte, retrospektive Auswertung von Patientendaten ausgestellt. Die Erhebung und Verarbeitung der Daten erfolgte unter Beachtung der gesetzlichen datenschutzrechtlichen Anforderungen und mit Zustimmung des Datenschutzbeauftragten des Universitätsklinikums Würzburg.

## Ergebnisse

Vom 01.01.2018 bis 31.10.2018 wurden 294 Patienten in den UICC Stadien III und IV im Kopf-Hals-Tumorzentrum Würzburg stationär behandelt. Im benannten Zeitraum wurden dem PD 75 stationäre Patienten (HNO: 67, MKG: 8) vorgestellt. Davon waren 73 (13 %) Patienten mit Kopf-Hals-Tumoren in den Stadien UICC III und IV, zwei weitere Patienten hatten ausgedehnte Lymphome im Bereich der großen Speicheldrüsen, sodass eine Einteilung nach UICC nicht möglich war. Das mittlere Alter lag bei 68 Jahren (Spannweite: 33–85 Jahre), der Großteil der Patienten war männlich (73 %). Ein Drittel der Patienten war zum Zeitpunkt des Erstkontakts mit dem PD tracheostomiert. Die Dauer zwischen Erstdiagnose und palliativer Erstvorstellung unterlag einer großen Spannbreite von einem Monat bis zu 10 Jahren (Median: 6 Monate, Tab. 1). Von der stationären Aufnahme bis zum Kontakt mit dem PD im Erhebungszeitraum betrug die Zeitspanne im Median 5 Tage (Spannweite: 0–85 Tage, Mittel: 12,4).

**Table 1:** 

Soziodemografie	*n* = 75 (%)
*Männlich*	55 (73 %)
*Weiblich*	20 (28 %)
*Diagnosen*	
C00–C14	59 (78 %)
C30–C32	16 (21 %)
*Tumorstadium*	
UICC III	8 (11 %)
UICC IV	65 (87 %)
Lymphome	2 (3 %)
Erkrankungsmonate seit Erstdiagnose (Mittelwert; Median; Spannweite; Standardabweichung)	54; 6; 0–1371; 22,8
*Tracheostomie*	
Vorhanden	25 (33 %)
Nicht vorhanden oder verschlossen	50 (67 %)

Vollständig hatten 34 der 75 Patienten den IPOS ausgefüllt (45 %). Die am häufigsten berichteten Symptome waren bei den physischen Belastungen „Schwäche“ (77 %), „Appetitlosigkeit“ (65 %) und „eingeschränkte Mobilität“ (65 %). „Sorgen um Erkrankung und Therapie“ sowie die „eigene Traurigkeit“, waren die emotionalen Belastungen mit der höchsten Häufigkeit (94 und 97 %). Von den Patienten gaben 85 % an, „nicht ausreichend informiert“ zu sein, und jeweils 77 % der Patienten konnten ihre „Gefühle nicht teilen“ und hatten „Probleme mit organisatorischen Belangen“ (Tab. 2).

**Table 2:** 

	0	1	2	3	4	≥2
*Physische Belastungen*						Prävalenz
Schmerz	12 (35)	8 (24)	8 (24)	4 (12)	2 (6)	14 (41)
Atemnot	14 (41)	16 (47)	3 (9)	1 (3)	0	4 (12)
Schwäche	1 (3)	7 (21)	9 (27)	14 (41)	3 (9)	**26 (77)**
Übelkeit	27 (79)	4 (12)	2 (6)	1 (3)	0	3 (9)
Erbrechen	32 (94)	1 (3)	0	1 (3)	0	1 (3)
Appetitlosigkeit	1 (3)	11(32)	14 (41)	7 (21)	1 (3)	**22 (65)**
Verstopfung	19 (56)	13 (38)	1 (3)	1 (3)	0	2 (6)
Mundtrockenheit	9 (27)	13 (38)	8 (24)	3 (9)	1 (3)	12 (35)
Schläfrigkeit	7 (21)	20 (59)	4 (12)	3 (9)	0	7 (21)
Eingeschränkte Mobilität	0	12 (35)	12 (35)	7 (21)	3 (9)	**22 (65)**
*Emotionale Belastungen*						
Sorgen des Patienten	1 (2,9)	1 (2,9)	10 (29,4)	11 (32,4)	11 (32,4)	**32 (94)**
Sorgen der Angehörigen	0	1 (3)	5 (15)	15 (44)	13 (38)	23 (68)
Traurigkeit	0	1 (3)	18 (53)	7 (21)	8 (24)	**33 (97)**
Innerer Frieden	1 (3)	7 (21)	12 (35)	13 (38)	1 (3)	26 (77)
*Kommunikative/praktische Bedürfnisse*						
Teilen von Gefühlen	1 (3)	7 (3)	12 (35)	10 (2)	4 (12)	**26 (77)**
Information	0	5 (15)	10 (29)	13 (38)	6 (18)	**29 (85)**
Organisatorisches	4 (12)	4 (12)	10 (29)	13 (38)	3 (9)	**26 (77)**

Nur unvollständig füllten 41 der 75 Patienten den IPOS-Bogen aus. Hierbei wurden die Fragen nach „ausreichender Information“ (43 %), dem „Teilen von Gefühlen“ und „organisatorischen Problemen“ (je 40 %), „Traurigkeit“ (39 %) und der „Besorgnis der Familie“ wie dem „inneren Frieden“ (je 37 %) am häufigsten nicht beantwortet.

Beim palliativem Erstkontakt lagen bei 59 Patienten (79 %) in den Patientenakten keinerlei Vorsorgedokumente vor. Bei 14 Patienten (19 %) wurde während der palliativmedizinischen Mitbetreuung eine Vorsorgevollmacht, bei 5 Patienten (7 %) eine Patientenverfügung und bei weiteren 8 Patienten (11 %) ein Notfallplan erstellt (Tab. 3).

**Table 3:** 

	Bei Aufnahme	Bei Tod oder Beendigung der Mitbetreuung
*Vorsorgevollmacht*		
Keine	59 (79 %)	45 (60 %)
Vorhanden und verfügbar	16 (21 %)	30 (40 %)
Davon gerichtliche Betreuung	2 (3 %)	–
*Patientenverfügung*		
Keine	66 (88 %)	61 (81 %)
Vorhanden und verfügbar	9 (12 %)	14 (19 %)
*PV als Notfallplan*		
Keiner	75 (100 %)	67 (89 %)
Vorhanden und verfügbar	0 (0 %)	8 (11 %)

Über 50 % der Patienten befanden sich in deutlich reduziertem Allgemeinzustand und waren zum großen Teil überwiegend bettlägerig und auf Hilfe angewiesen (ECOG 4 und 5; Tab. 4).

**Table 4:** 

ECOG-Performance Status	*n* = 75 (%)
*1 (normale Aktivität)*	3 (4)
*2 (leichte Arbeiten möglich)*	7 (9)
*3 (<50* *% des Tages im Bett)*	21 (28)
*4 (>50* *% des Tages im Bett)*	27 (36)
*5 (permanent bettlägerig)*	17 (23)

*ECOG* Eastern Cooperative Oncology Group

Insgesamt verstarben im Beobachtungszeitraum 19 Patienten mit Kopf-Hals-Tumoren innerklinisch. Alle 19 Patienten (100 %) wurden vom PD mitbetreut. Hiervon verstarben 5 Patienten (26 %) auf der Intensivstation, 8 Patienten (42 %) auf der Normalstation und 6 Patienten (32 %) auf der Palliativstation. Von den an unserem Klinikum verstorbenen Patienten hatten 13 (68 %) bereits 3 Tage und länger vor ihrem Tod Kontakt zur Palliativmedizin. Lediglich 6 verstorbene Patienten (32 %) hatten erst weniger als 3 Tage vor ihrem Tod Kontakt zur Palliativmedizin. Somit wird der Earle-QI, dass höchstens 8 % der Patienten ihren palliativen Erstkontakt weniger als 3 Tage vor dem Tod haben sollen, nicht erreicht.

## Diskussion

### Symptomlast

#### Physische Belastungen

Obwohl Schmerz bei Patienten mit Kopf-Hals-Tumoren ein hochrelevantes Symptom ist [15], zeigte sich bei der Erfassung mit dem mehrdimensionalen IPOS bei den hier befragten Patienten ein anderes Bild. Schmerzen waren bei unseren Patienten weit weniger vorhanden, als Bossi et al. berichten (42 vs. 70 %) [15]. Eine mögliche Erklärung ist die vorhandene schmerztherapeutische und zunehmend wachsende palliativmedizinische Kompetenz der primär behandelnden Teams. Da die Patienten im Median erst 5 Tage nach ihrer stationären Aufnahme dem PD vorgestellt wurden, konnte die erste Symptomlast oft bereits vom primären Behandlungsteam therapiert werden und wurde somit durch den IPOS nicht mehr erfasst. Wie Murtagh et al. 2019 [6] zeigen konnten, ist der IPOS ein sehr veränderungssensibles Instrument. Da auch in der von uns verwendeten Patientenversion ganz konkret die Symptome der letzten 3 Tage abgefragt wurden, erklärt dies, warum schnell behebbare Ursachen nicht erfasst wurden. Im Gegensatz hierzu wurden diejenigen Symptome gut erfasst, die sich bei fortgeschrittener Erkrankung nur schlecht oder zögerlich verbessern lassen. Hierzu gehören, wie auch Murtagh et al. und Bossi et al. [6, 15] berichten, „Schwäche“, „zunehmende Immobilität“ und „Appetitlosigkeit“. Insgesamt sind dies alles Zeichen einer voranschreitenden Grunderkrankung und spiegeln sich auch in den Stadien des ECOG Performance Status wider, da sich 59 % unserer Patienten im Stadium ECOG 4 oder 5 befanden.

#### Emotionale Belastungen

Über „Traurigkeit“ berichten 97 % der Patienten. Bereits Lydiatt et al. [16] konnten zeigen, dass die Rate an Depression und Suiziden bei Patienten mit Kopf-Hals-Tumoren sehr hoch ist. Deshalb sollte mit der palliativmedizinischen Beratung auch eine regelhafte und frühzeitige psychoonkologische Mitbetreuung angeboten werden [5, 17].

#### Kommunikative und praktische Bedürfnisse

Ein Drittel der Patienten war tracheostomiert, was mit kommunikativen Defiziten und einem zusätzlichen Unterstützungsbedarf einhergehen kann [18, 19]. Auch das „Teilen von Gefühlen“, welches für 77 % der Patienten ein Problem darstellt, kann durch die fehlende oder eingeschränkte Option zu sprechen zusätzlich erschwert sein [20].

Durch die körperlichen Veränderungen stehen die Patienten häufig zusätzlich vor existenziellen organisatorischen Problemen, wie der Versorgung eines Tracheostomas oder der Anpassung der häuslichen Situation an die veränderten Lebensumstände. Über entsprechende „organisatorische Probleme“ berichten ebenfalls 77 % unserer Patienten.

Durch die Erfassung der belastenden Symptome mittels PROM kann der Patient Belastungen und eventuell selbst nicht erkannte Bedürfnisse einfacher äußern. Auch wenn derzeit noch klare Cut-offs im Hinblick auf den IPOS-Summenscore fehlen, bietet er einen guten Anhaltspunkt für die mögliche Notwendigkeit einer spezialisierten palliativen Mitbetreuung [6].

### Vorsorgedokumente

Ein Großteil der Patienten (77 %) hatte beim Erstkontakt mit dem PD keine Vorsorgedokumente verfügbar. Wenn Dokumente vorhanden waren, so handelte es sich häufiger um VV als um PV. Die Nachfrage nach Vorsorgedokumenten gehört zum standardisierten Basisassessment bei palliativem Erstkontakt. Eine Beratung wird allen Patienten, auch im Beisein Angehöriger, angeboten und stellt einen wesentlichen Bestandteil dar [21]. Eine frühzeitige und bei Veränderungen immer wiederkehrende Diskussion über eine vorausschauende Planung am Lebensende ist empfehlenswert [3, 5]. Die Erklärung, was Vorsorgedokumente sind und wie hilfreich diese sein können, sind häufig der Türöffner für weitere Gespräche [22, 23]. Die Vorteile, die eine vorausschauende Planung zum Lebensende bietet, sind gut bekannt: So kann die Patientenzufriedenheit am Lebensende gesteigert werden und auch Angst und Depression bei den hinterbliebenen Angehörigen gemindert werden [9].

Insgesamt war die Bereitschaft, eine VV während eines stationären Aufenthalts zu erstellen, deutlich höher, als eine PV zu erstellen. Insbesondere dann, wenn Patienten sich über die Behandlungswünsche am Lebensende noch keine Gedanken gemacht haben, kann es sinnvoll sein, zunächst einen Vorsorgebevollmächtigten zu bestimmen und auf eine PV zu verzichten [22]. Dies schafft Zeit und bietet den Vorsorgebevollmächtigtem und dem Vollmachtgeber die Chance, miteinander ins Gespräch zu kommen. Für Patienten ist es wichtig, zu klären, wer im Fall der eigenen Einwilligungsunfähigkeit auskunfts- und entscheidungsbevollmächtigt ist. Hiermit wird dem Arzt im Hinblick auf die Legitimation ärztlicher Handlung ein wertvoller Partner zur Seite gestellt.

Bei der Erstellung einer Patientenverfügung anhand von standardisierten Vorlagen besteht häufig das Problem, dass die Situationsbeschreibungen in diesen standardisierten Patientenverfügungen die individuelle Situation von fortgeschritten erkrankten Patienten nicht richtig erfassen. Insbesondere für Kopf-Hals-Tumor-Patienten mit ihren therapieassoziierten Problemen, wie Tracheostoma, Lymphödem, Ernährungs- oder Kommunikationsschwierigkeiten, trifft dies gehäuft zu.

Auch nach ausführlicher Beratung erstellten lediglich 5 Patienten (7 %) während des stationären Aufenthalts eine PV. Dies ist bedauerlich, da der Behandler ohne eine Patientenverfügung, im Hinblick auf den Patientenwillen ggf. auf die Aussagen von Vorsorgebevollmächtigten oder anderen Vertrauenspersonen angewiesen ist. Oftmals sind es aber auch die Angehörigen, die Diskussionen über das Lebensende vermeiden wollen, bzw. haben manche der Patienten gar keine Angehörigen, mit denen sie diese Themen besprechen könnten [24]. Häufig werden Wünsche am Lebensende gar nicht an Vorsorgebevollmächtigte kommuniziert und auch nicht konkret in einer PV festgehalten [25]. Gerade in diesen Gesprächen können jedoch Patienten und Angehörige erkennen, was ihnen wichtig ist, und dabei helfen, dieses an das Behandlerteam zu kommunizieren und somit eine Therapie zu erhalten, die den eigenen Wünschen und Werten entspricht [9–11].

### Notfallplan

Insgesamt wurden mit 8 Patienten Notfalldokumente erstellt. Hierbei wurden den Patienten alle möglichen und auch zu erwartenden Komplikationen in gleichem Maße wie realistische und medizinisch sinnvolle Therapieoptionen ausführlich aufgezeigt [13]. In der Therapieentscheidung hat der Patient im Rahmen des medizinisch Sinnvollen ein klares Mitspracherecht. Alle Patienten, die mit uns während des stationären Aufenthalts ein Notfallplan erstellten, entschieden sich für symptomlindernde Maßnahmen in der Häuslichkeit. Eine Verlegung in ein Krankenhaus war nur dann gewünscht, wenn Symptome im Versorgungsumfeld nicht beherrschbar waren. Eine eventuelle Lebensverlängerung vor dem Hintergrund des medizinisch Möglichen [26] war von diesen 8 Patienten nicht gewünscht.

Ein solches Vorgehen, und insbesondere das Vorhandensein einer klaren Handlungsanweisung für den Notfall, gibt nicht nur dem Patienten und den Angehörigen, sondern auch dem Behandler Sicherheit in der Akutsituation [27]. Hierbei sollen nicht nur die Behandlungswünsche des Patienten am Lebensende erfasst werden, sondern letztlich realistische Therapieoptionen mit allen Beteiligten, insbesondere mit den behandelnden HNO/MKG-Ärzten, Schritt für Schritt entwickelt werden [3, 5, 13].

Patienten benötigen häufig Zeit, um mit veränderten Ausgangsvoraussetzungen umzugehen. Ein einmaliger Kontakt zur Palliativmedizin und eine einmalige Beratung zur vorausschauenden Planung ist häufig nicht ausreichend [22]. Das zeigt sich auch daran, dass die Patienten in unserem Kollektiv im Mittel 1,7 (Median: 2; Spannweite: 1–4) angeforderte Konsile zur palliativmedizinischen Mitbetreuung hatten.

### Qualitätsindikator

Der von Earle definierte Qualitätsindikator wurde im Hinblick auf die Betreuungsdauer/Kontaktzeit vor dem Versterben nicht erreicht (32 % Kontaktzeit zum PD < 3 Tage vor Tod am UKW). Hier ist Verbesserungspotenzial erkennbar. Den Gründen für die späte Zuweisung zur Palliativmedizin soll in Fallanalysen weiter nachgegangen werden. Möglicherweise tragen auch finale Noteinweisungen von Kopf-Hals-Tumor-Patienten ohne Mitbetreuung durch eine SAPV (spezialisierte ambulante palliative Versorgung) zu den kurzen stationären Palliativkontaktzeiten bei.

#### Limitationen

Wir untersuchten retrospektiv eine selektionierte Patientengruppe an einem Haus der Maximalversorgung. Auf andere Kliniken sind diese Ergebnisse nur bedingt übertragbar. Perspektivisch wäre ein standardisiertes Screening auf Palliativbedarf bei allen Tumorpatienten im Stadium der Unheilbarkeit frühzeitig wünschenswert.

## Fazit für die Praxis

Die am häufigsten geäußerten, belastenden Symptome der vom Palliativdienst betreuten Patienten mit Kopf-Hals-Tumoren in inkurabler Situation sind weniger physischer, sondern emotionaler und organisatorischer Natur. Dies ist ein klarer Hinweis auf die guten Kompetenzen der Primärbehandler bezüglich einer notwendigen Symptomlinderung. Insofern sollte der Schwerpunkt eines PD eher im psychosozialen und vorausschauend planerischen Bereich liegen. Hierzu gehört auch das Angebot zur Versorgungsplanung und zur Erstellung von Vorsorgedokumenten.
